# Dermatological Notes

**Published:** 1889-03

**Authors:** M. B. Hutchins

**Affiliations:** New York


### SOME DERMATOLOGICAL NOTES.

*To the Editors Atlanta Medical and Surgical Journal :*

Like every other branch of the practice of medicine, that of Dermatology is constantly undergoing changes involving anatomy (microscopic), physiology, diagnosis and treatment. One by one the "skin men" of New York are accepting the views advanced by Unna, of Hamburg, upon the new form of skin disease and its cause. It is a new form only in having received its definite designation, and in the fact of its cause being at last recognized. The new element considered is the *seborrhœal*, and we have descriptions of *seborrhœal eczema*, principally, and other diseases which are designated occasionaly as *seborrhœal;* as "*seb. rosacæ, seb. roseola*," and "*sypihlis on a seborrhœic base.*" This discovery is interesting in that it presents us to an old acquaintance which is nearly universal—dandruff—especially the dirty, greasy, crusty form, so commonly seen in people of oily skins. "Eczema Seborrhoicum" is the most prominent and most common. Three forms are observed: the scaly, the crusty and the weeping, or moist, and the disease, as noticed upon the face, around the ears, (and on the scalp,) on the chest, arms, and in the axillæ, may generally partake of either of these forms, though the "weeping" form is more common in the axillæ and the "crusty" over the sternum. There is nearly always a history of progession downward from the scalp. The patches tend to circumscription, present little infiltration, and there is a marked oiliness, or greasy feature, in the products of the disease. The scabs produced are yellow, thin and greasy; itching not usually so severe as in other eczemas. Unna places sulphur in oxide of zinc ointment, first in the treatment of the moist forms; resorcin, pyrogallol and chrysorobin in the scaly or crusty varieties. The usual forms are best treated with resorcin. A 3 per cent. aqueous and alcoholic lotion of resorcin is applied every morning, and at night 4 per cent. resorcin in ointment. The first indication for treatment is in the condition of the scalp, this being treated principally. It is claimed that there is only a minimum chance of the occurrence of seborrhœal disease if the scalp is kept in a healthy condition.

Another point of interest in this affection is the new function attributed to the coiled, or sweat, glands. We now hear—among the followers of German teaching—of the "so-called sweat or fat producing glands of the skin." It is claimed that, during an attack of *eczema seborrhoicum* the sebacious follicles are plugged up, that the disease may occur on the palms where there are no sebacious glands and that the oil, or fat, upon the surface is identical with that found in the sweat canals and coiled glands of the skin. There is an established theory that the sweat is exuded into the canals from the lymph-spaces, while the function of the coiled glands is that of producing the oil of the skin.

Another recent innovation in Dermatology comes from the same German author, and is being generally tried here. It is the local application of medicament in the form of plasters, containing a given per cent. of the drug, uniformly mingled with a certain per cent. of adhesive material—something like the old belladonna plaster, but made with greater exactitude, and so that the medical ingredient is all available. These plasters afford constant, close application of the remedy, are convenient, and cleaner than ointments or oils. As made here they are imperfect, perhaps, but there is open a great field for their successful use.

Perhaps one of the most chronic cases of eczema on record is that of a patient now in the Skin and Cancer Hospital. The woman is 44 years old, and states that she has never been entirely free from the disease since the age of two months. Her general health is "fair," and the eczema is improving under treatment.

Anthrarobin, a new drug, is gaining friends as an application in cases of *psoriasis*. When chrysorobin and pyrogallic acid are indicated, acting well upon the diseased spots without the irritation of the adjacent skin of the former, or the diffuse staining and production of scales or follicalitis, upon the adjacent skin, in the use of the latter. It is generally used in 10 per cent. strength.

A rather unusual localisation of *chloasma* was seen in a patient; a woman—who applied for treatment in January. Patient was 38 years old. Extensor surfaces of both fore-arms and wrists were deeply pigmented, pigmentation being diffuse, though there were a few isolated "spots." History of occurrence upon forehead, when pregnant; and of scant menstruation for the twelve months previous to the time of visit to the clinic; and swelling of face and hands noticed in the morning. Resorcin plaster was applied, and patient has not since been seen.

Two patients applied during January for treatment—thinking they had "cancer" of the lip. Each case was diagnosed "cancer of the lip," and the previous history or subsequent developments sustained the diagnosis—while the disease rapidly declined under treatment.

A man came into the clinic with a large crusted lesion, somewhat like syphilitic rupia under each orbit. He had been taking *salicylate* of *sodium*, and the lesions were diagnosed as produced by the drug. Later suppuration occurred beneath the crusts. Continued administration of the drug failed to produce other lesions.

I asked one of the physicians of the *cancer* department of the hospital, for the percentage of "cures" in all forms and stages of "cancer," and, if I remember precisely, he answered: "Of all—about 17 per cent. uterine the most unfavorable, being 3 per cent., while those of the lower lip offer the best prospect of permanent cure." A case is now in the hospital dying of a return of the disease in the pelvic and vaginal tissues, which occurred one month after total extirpation of the uterus last fall, and previous to her recent admission here. Chian turpentine—in about five grain doses—either in solution or capsules, has been thoroughly tried here, with doubtful effect upon the disease, while the unpleasant effects upon the stomach and pains in the head, render its use difficult.

Ichthyol as a stimulant and antiseptic in indolent ulcerations, or in certain forms of eczema, as in indications for tar, has been seen to have a fine effect. It is used in 3 per cent. to 10 per cent. strength, according to indications.

A leading New York Dermatologist, who makes a point of treating the digestive system in skin diseases, in addition to the local applications, is quite an advocate of the following combination of old ingredients: Pil. Hydrarg., Ext. Colocynth. Co. *āā* gr. iiss., Pulv. Ipecac, gr. ss., and claims that their action previous to a voyage will prevent sea-sickness.

M. B. HUTCHINS, M. D.

*New York, February 1889.*

---

SULPHONAL.—A Berlin correspondent writes to the *Medical and Surgical Reporter* that Dr. Bornemany has reported a case of severe poisoning from the administration of sulphonal. The chief symptoms were incoördination in the movements, first in the lower extremities and later in the arms, which vision did not seem to affect, and illusions and hallucinations. The drug did not seem to exert any unfavorable influence over the circulation.

